# *Hedysarum coronarium-Based* Green Composites Prepared by Compression Molding and Fused Deposition Modeling

**DOI:** 10.3390/ma15020465

**Published:** 2022-01-08

**Authors:** Roberto Scaffaro, Maria Clara Citarrella, Emmanuel Fortunato Gulino, Marco Morreale

**Affiliations:** 1 Department of Engineering, University of Palermo, Viale delle Scienze, ed. 6, 90128 Palermo, Italy; mariaclara.citarrella@unipa.it (M.C.C.); emmanuelfortunato.gulino@unipa.it (E.F.G.); 2 Faculty of Engineering and Architecture, Kore University of Enna, Cittadella Universitaria, 94100 Enna, Italy

**Keywords:** green composites, biocomposites, FDM, biopolymers, Mater-Bi, natural filler, additive manufacturing, 3D printing

## Abstract

In this work, an innovative green composite was produced by adding *Hedysarum coronarium* (HC) flour to a starch-based biodegradable polymer (Mater-Bi^®^, MB). The flour was obtained by grinding together stems, leaves and flowers and subsequently sieving it, selecting a fraction from 75 μm to 300 μm. Four formulations have been produced by compression molding (CM) and fused deposition modeling (FDM) by adding 5%, 10%, 15% and 20% of HC to MB. The influence of filler content on the processability was tested, and rheological, morphological and mechanical properties of composites were also assessed. Through CM, it was possible to obtain easily homogeneous samples with all filler amounts. Concerning FDM, 5% and 10% HC-filled composites proved also easily printable. Mechanical results showed filler effectively acted as reinforcement: Young’s modulus and tensile strengths of the composites increased from 74.3 MPa to 236 MPa and from 18.6 MPa to 33.4 MPa, respectively, when 20% of HC was added to the pure matrix. FDM samples, moreover, showed higher mechanical properties if compared with CM ones due to rectilinear infill and fibers orientation. In fact, regarding the 10% HC composites, Young’s modulus of the CM and FDM ones displayed a relative increment of 176% and 224%, respectively.

## 1. Introduction

In recent years, there has been increasing attention to biopolymeric systems containing plant-based biomass. Combining biodegradable polymers with agricultural waste, marine waste or with industrial residues from wood processing, has gained particular interest in the view of producing composite materials with high mechanical performance [1,2,3,4,5]. Green composites, moreover, are considered an effective strategy for the reduction of environmental pollution related to plastic [6,7,8,9].

The most commonly used biopolymeric matrices employed to obtain green composites are PLA, PCL, PBAT, cellulose and starch-based ones [1,6]. Mater-Bi^®^ (MB) is a family of commercial starch-based biopolymers that finds relevant applications in many fields thanks to its interesting mechanical properties, good thermal stability, excellent processability, full biodegradability and suitability to be reinforced with natural fibers, as reported in many studies [4,5,7,10]. The addition of an organic filler to these matrices could accelerate their biodegradability [6,7] while enhancing mechanical performance [1,7,11,12,13,14]. Potentially, all plant-based biomasses present peculiar characteristics that may help achieving both goals.

The Mediterranean area offers a great variety of plant species that can find applications in the preparation of biodegradable polymer-based composites. The addition of two different amounts (10% and 20%) of Opuntia Ficus Indica flour to poly(lactic acid) (PLA), for the production of composites by compression molding, led to an increase in the elastic modulus on increasing filler content [11]. Several studies investigated the structure–property relationships of biocomposites obtained by adding Posidonia Oceanica leaves to different biopolymers, verifying its ability to enhance mechanical properties of the pure polymeric matrix [10,12,13]. Moreover, the addition of Posidonia Oceanica to biopolymers as a filler can accelerate their degradability [15,16]. It has been reported that adding 15% of cellulose fibers extracted from Mediterranean saltbush (Atriplex halimus) to PLA matrix raised the tensile strength and Young’s modulus by 25% and 45%, respectively, while the elongation at break was sensibly reduced [14]. Therefore, the current challenge is to investigate new natural fillers that should be easily available, inexpensive, and abundantly present on the territory.

Hedysarum coronarium (HC) is a fodder, a watery herbaceous plant rich in soluble sugars, consisting of a very dense and structured root system. This characteristic makes it unique among leguminous plants in its ability to penetrate and grow spontaneously in many types of soil. It is very abundant in the Mediterranean region and constitutes significant trouble due to its tendency to accumulate on countryside areas. It is considered a weed grass, and for this reason, it must be collected and disposed of. HC is currently used in the agri-food sector but, to our best knowledge, was never used before to prepare green composites. Since HC is constituted by a very branched, hollow and fistulous stem, the addition of this plant to a polymeric matrix could be very interesting in order to improve the mechanical performance while preserving the biodegradability/compostability of the composite. Moreover, the addition of a certain amount of natural scraps to a polymeric matrix reduces the costs of the final product [17,18].

Typically, thermoplastic-based green composites are produced by compression molding, extrusion or injection molding [19]. However, in recent years, fused deposition modeling (FDM) has been investigated as an alternative for green composite processing [20,21,22,23,24,25]. In fact, among the many advantages of FDM, the reduction of production time and costs, as well as the possibility to create extremely elaborated geometries, make this production technique one of the most promising for the creation of manufactured items based on green composites [25].

In this study, an innovative green composite was produced for the first time by adding HC flour to a biodegradable polymer (Mater-Bi^®^). The dried HC stems, leaves and flowers were ground and sieved. Four formulations were produced by melt mixing 5%, 15%, 10% and 20% of HC to MB. The resulting biocomposites were then employed for the realization of samples both for CM and FDM. Rheological, morphological and mechanical characterizations were carried out on the starting materials and the biocomposites. The final goal of this study is to replace up to 20% of bioplastic with low-cost and ecofriendly natural filler while enhancing mechanical performance.

## 2. Materials and Methods

### 2.1. Materials

HC (*Hedysarum coronarium* L., syn. *Sulla coronaria* [L.] Medik. Species Sparacia) used in this study were kindly supplied by “Azienda Agricola Alberto Lo Dico” Petralia Soprana (PA), Sicily, Italy and successively washed and dried in a vacuum oven (NSV9035, ISCO, Milan, Italy) at T = 40 °C for 3 days and ground. The plants were about 40 cm high when mowed. From the visual inspection of the plant, thanks to the characteristic appearance of the flower, it was found that the biomass received was made up entirely of Hedysarum coronarium. In this study, the whole plant was ground as received in order to optimize production time and costs.

HC dried steam showed a Young’s modulus of 1545 MPa, a tensile strength of 23.3 MPa and an elongation-at-break of 20.3%. The flour obtained by grinding the whole plant together displayed an average density of 1.6 g/cm^3^.

Mater-Bi^®^ EF51L (MB), supplied by Novamont SpA (Novara, Italy) is a film extrusion grade biopolymer based on blends of aliphatic and aromatic biodegradable co-polyesters with proprietary composition. MB was vacuum-dried overnight at T = 60 °C in order to prevent polymer hydrolytic scission during processing.

### 2.2. Preparation of Composites

Firstly, the dried HC was ground for 3 min and then sieved in order to obtain particles of a size suitable for the 3D printer (Next Generation, Sharebot, Nibionno, Italy), which, therefore, do not lead to obstructions in the nozzle. To this aim, and considering previous studies [11,20], the sieving fraction from 75 μm to 300 μm was selected. Prior to processing, the obtained HC flours and MB pellets were dried overnight in a vacuum oven (NSV9035, ISCO, Milan, Italy) at 40 °C and 60 °C, respectively.

In order to obtain a homogeneous dispersion of the filler, according to previous studies [20], the filler amounts chosen to prepare MB-based biocomposites were 5, 10, 15 and 20 wt%. All of the composites (namely MB/HC5, MB/HC10, MB/HC15, MB/HC20) and neat MB, for comparison, were prepared by melt compounding in a internal mixer (Plasticorder, Brabender, Duisburg, Germany; T = 170 °C, rotor speed = 64 rpm, t = 5 min).

The obtained materials were then ground into pellets and processed in a Polylab single-screw extruder (Haake Technik GmbH, Vreden, Germany; L/D = 25; D = 19.05 mm), operating at 40 rpm screw speed and 140-150-160-170 °C temperature profile. The extrudates were drawn with the help of a conveyor belt system (take-up speed = 5.5 m/min), to obtain filaments with a diameter suitable to the printer (1.75 mm).

The compression-molded samples (CM) were obtained using a laboratory press (Carver, Wabash, IN, USA) at 160 °C and 180 bar for 2 min. The samples were finally cut into specimens of appropriate geometry (60 mm × 10 mm) for further characterizations.

The samples obtained for fused deposition modeling (FDM) were first designed with the help of CAD Solid Edge 2019^®^ software (Plano, TX, USA), and the STL files produced were elaborated on Simplify3D^®^ software (Cincinnati, OH, USA) to obtain the gcode files. For each formulation, 60 mm × 10 mm × 1 mm samples were printed using a Sharebot Next Generation (Nibionno, Italy) 3D printer. FDM operating parameters are reported in Table 1. Nozzle temperature was chosen after some trials, aiming to avoid nozzle obstructions and to obtain good printability performance. The other parameters were chosen based on the scientific literature [20,26,27,28]. In particular, a 100% infill rate and a rectilinear infill pattern with a 0° raster angle were chosen in order to optimize the tensile properties [24]; 45 mm/s printing speed was chosen as a compromise between better properties and higher production rate.

**Figure 1:**
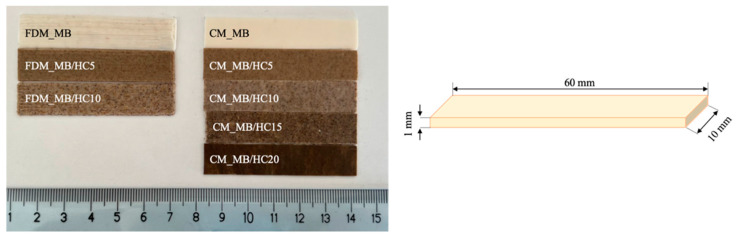
FDM- and CM-obtained samples.

Sample formulations and their code names are reported in Table 2. Moreover, some representatives of obtained CM and FDM samples are shown in Figure 1.

### 2.3. Rheological Characterization

Rheological properties of the samples were analyzed, using a rotational rheometer (ARES- G2, TA Instruments, New Castle, PA, USA) equipped with a 25 mm parallel-plate geometry. All the tests were performed at 160 °C, in frequency sweep mode in the range 1–100 rad/s, by imposing a constant stress of 1 Pa.

### 2.4. Morphological Analysis

The morphology of CM and FDM samples was observed by using a scanning electron microscope (Phenom ProX, Phenom-World, Eindhoven, The Netherlands) with optical magnification range of 20–135×, electron magnification range of 80–1.3 × 10^5^, maximal digital zoom of 12×, and acceleration voltages of 15 kV. The microscope is equipped with a temperature controlled (25 °C) sample holder. The samples were fixed on an aluminum stub (pin stub 25mm, Phenom-World, Eindhoven, The Netherlands) using a glued carbon tape.

### 2.5. Mechanical Characterization

The mechanical behavior of the samples was investigated by tensile tests, carried out using a laboratory dynamometer (mod.3365, Instron, Norwood, MA, USA) equipped with a 1 kN load cell. The tests were performed on rectangular-shaped specimens (60 mm × 10 mm). The measurements were performed by using a double crosshead speed: 1 mm min^−1^ for 2 min and 50 mm min^−1^ until fracture occurred. The grip distance was 30 mm, whereas the sample thickness was measured before each test. Eight specimens were tested for each sample, and the results for elastic modulus (E), tensile strength (TS) and elongation at break (EB) have been reported as average values ± standard deviations.

## 3. Results and Discussion

Neat MB and HC composite filaments were easily extruded with a diameter suitable for the printer (1.75 mm), and part of them were pelletized and processed by compression molding. Through CM, it was possible to obtain homogeneous samples with all filler amounts. Regarding the FDM samples’ printability, pure MB and the ones containing 5% and 10% of HC proved easily printable once the appropriate parameters were found. The biocomposite containing more than 10% of HC, in fact, could not be produced by FDM with the printer used in this study. In detail, 15% and 20% of HC-containing filament does not even flow in the melting chamber.

**Figure 2:**
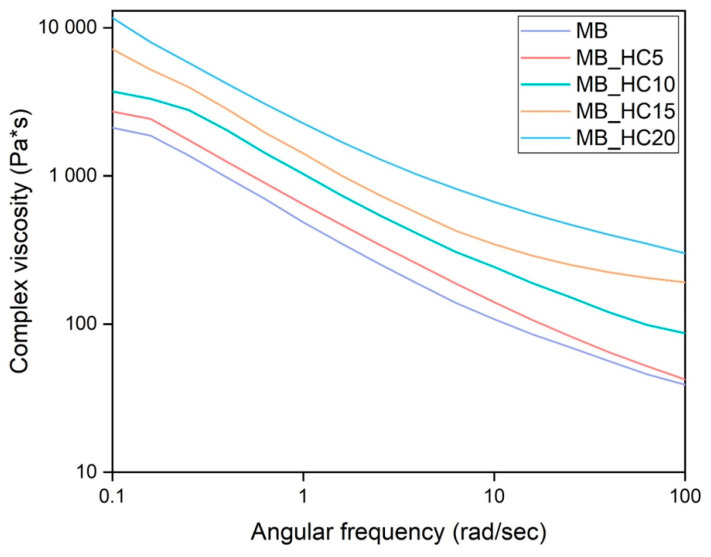
Complex viscosity of pure MB and its HC composites.

It is known from the scientific literature that rheological properties of the polymeric filament have a strong influence on printability [29]. For this reason, rheological properties of biocomposites have been investigated. In Figure 2, the rheological curves of MB and its HC composites are shown. For MB, a pseudoplastic behavior was generally observed, with remarkable shear thinning at higher frequencies. In general, the addition of HC leads to a variation of polymer viscosity: when increasing filler content, the viscosity values monotonically increase in the entire investigated frequency range. It is worth observing that MB/HC15 and MB/HC20 show a remarkably non-Newtonian behavior with the presence of apparent yield stress in the low frequency region, in agreement with the findings on other similar composites [30]. This behavior could reasonably justify the nonprintability of 15% and 20% of HC-containing composites. As soon as filament reaches the melting chamber, in fact, the very first part of the filament melts and, due to its high viscosity, creates a plug at the entrance of the chamber that clogs it, not allowing the filament to advance. Therefore, filaments do not melt in the chamber, leaving it empty and thus preventing the sample printing.

**Figure 3:**
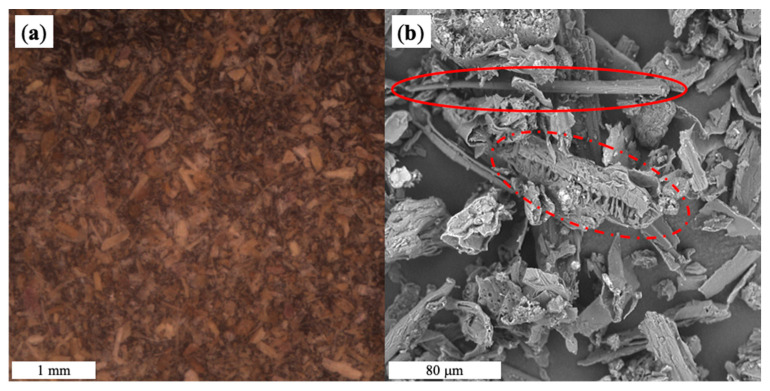
Optical image (**a**) and SEM micrograph (**b**) of HC powder.

Morphology of MB samples, HC powder and obtained composites were analyzed by SEM. Relevant optical image and micrograph of the natural organic filler are shown in Figure 3. From SEM micrograph (Figure 3b), it is possible that the HC powder contains elements with different morphology, reasonably belonging to different parts of the plant: the solid line highlights a part probably belonging to the stem, while the dashed line highlights a flower.

**Figure 4:**
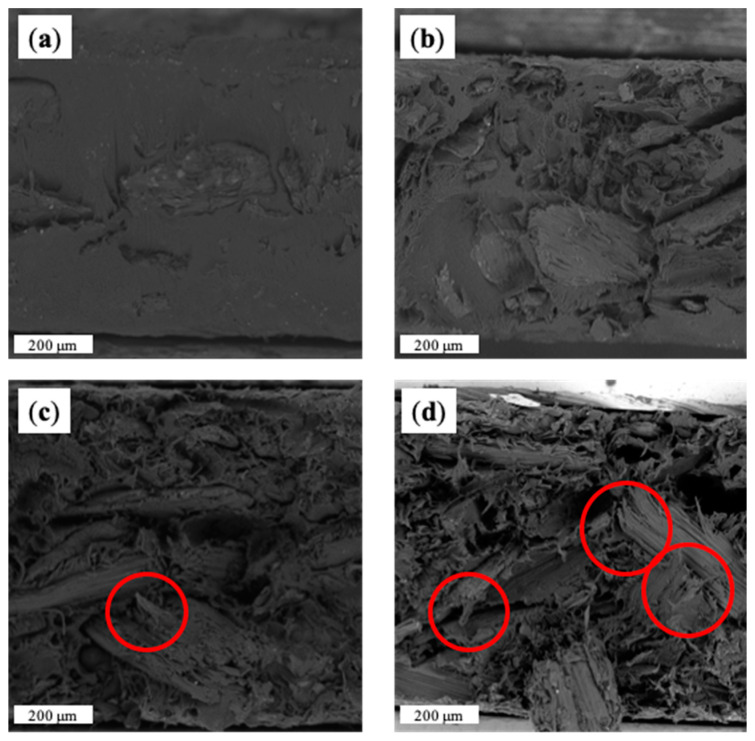
SEM micrograph of fractured cross-sections of CM_MB/HC5 (**a**), CM_MB/HC10 (**b**), CM_MB/HC15 (**c**) and CM_MB/HC20 (**d**). Red circles highlight poor filler wettability parts.

Morphological analysis was also performed on the transverse fracture surfaces of CM samples, for which relevant SEM micrographs are reported. In Figure 4, it is possible to notice an almost homogeneous dispersion of the filler in each CM sample. In addition, matrix–filler adhesion appears to be good for all CM series. However, as filler content increases, the wettability of the matrix decreases. In particular, when the filler level is 15 wt%, the matrix is not able to homogenously wet the filler, as the red circle in Figure 4c clearly highlights. This behavior is obviously more remarkable when the 20% HC is added (red circle, Figure 4d). As evidence, poor wettability does not prevent the production of specimen for CM, whereas, on the contrary, the FDM printability of 15% and 20% of HC-containing samples was totally inhibited.

**Figure 5:**
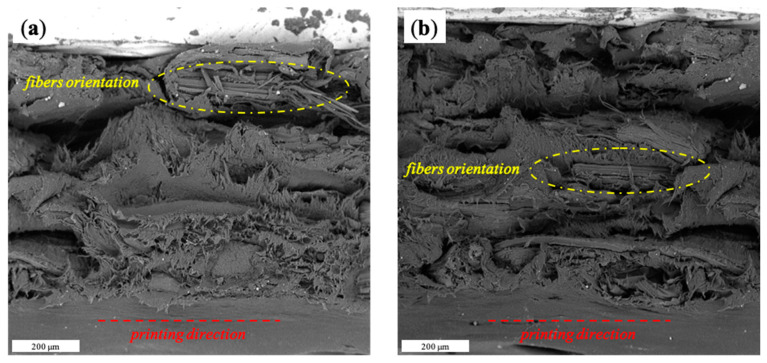
SEM micrograph of fractured cross-sections of FDM_MB/HC5 (**a**); FDM_MB/HC10 (**b**).

Mechanical performance of samples was investigated by tensile tests. The values of elastic modulus (E), tensile strength (TS) and elongation at break (EB) of neat MB and MB composites with different amount of HC produced via CM and FDM are reported in Table 3. Regarding CM samples, CM_MB showed a Young’s modulus of 74.3 MPa a tensile strength of 18.6 MPa and an elongation-at-break of 821%. As the filler content increased, Young’s modules and tensile strengths of the composites increased too. On the contrary, EB decreased significantly when the filler was added to the matrix. CM_MB/HC20 exhibited a Young’s modulus value of 236 MPa while the EB value dropped to 20.3%. Concerning FDM composites, the same trend was observed. In particular, FDM_MB showed a Young’s modulus of 83.9 MPa. When 5% or 10% filler amount was added, Young’s modulus values of the composites reached 166 MPa and 188 MPa, respectively. It is well known from the scientific literature, in fact, that the addition of organic fillers to a biopolymeric matrix typically brings an enhancement of the mechanical properties [31]. Moreover, the FDM prepared samples showed a more remarkable improvement of the mechanical performance if compared to the ones obtained by CM. Regarding the 10% HC composites, in fact, Young’s modulus of CM and FDM samples displayed a relative increment of 176% and 224%, respectively. This behavior could be reasonably ascribed to 100% rectilinear infill [24,26]. Moreover, SEM micrograph of FDM_MB/HC5 (Figure 5a) and FDM_MB/HC10 (Figure 5b) showed that, during the printing process, HC fibers aligned along printing orientation. This fiber alignment could reasonably improve the mechanical properties of the FDM composites [32].

## 4. Conclusions

An innovative green composite was produced for the first time by adding 5%, 10%, 15% and 20% of *hedysarum coronarium* (HC) flour to Mater-Bi^®^. Through CM it was possible to obtain homogeneous samples with all percentages of fillers. Moreover, neat MB and the samples containing 5% and 10% of HC proved easily printable too. The findings of this study outline that it is possible to replace up to 20% of bioplastic with a low-cost and ecofriendly organic filler, enhancing the mechanical performance at the same time. Mechanical results, actually, showed that filler effectively acted as a reinforcement: Young’s modules and tensile strengths of the composites increased from 74.3 MPa to 236 MPa and from 18.6 MPa to 33.4 MPa, respectively, when 20% of HC was added to the pure matrix. FDM samples, moreover, showed higher mechanical properties if compared with CM ones due to rectilinear infill and fibers orientation. In fact, regarding the 10% HC composites, Young’s modulus of CM and the FDM ones displayed a relative increment of 176% and 224%, respectively. The improvement of the mechanical properties together with other advantages of FDM, such as reduction of production times and costs, and the possibility to produce extremely elaborated geometries, make MB/HC promising for the creation of manufactured products based on green composites that, thanks to their wood-like color, can find application in green fabrication of panels for different use, in the structural sector, in the production of furniture and so on. In the scientific literature, indeed, other similar systems (biodegradable polymeric matrices and natural fillers) are already proposed for these types of applications. Panels for sound adsorption, thermal insulation or for the interior design industry, in fact, do not require extremely high mechanical properties [20,33,34,35,36].

## Figures and Tables

**Table 1:** FDM process parameters.

FDM Operating Parameter	Value
Nozzle temperature	160 °C
Bed temperature	60 °C
Infill rate	100%
Infill pattern	Rectilinear
Raster angle	0°
Layer thickness	0.1 mm
Extrusion width	0.4 mm
Printing speed	50 mm/s
Perimeter shells	1
Sample Orientation	flat

**Table 2:** Formulation of investigated samples.

Sample Code Name	MB Content (wt%)	HC Content (wt%)	HC Mesh Size (μm)	Production Technique
CM_MB	100	0	-	CM
CM_MB/HC5	95	5	300 < 75	CM
CM_MB/HC10	90	10	300 < 75	CM
CM_MB/HC15	85	15	300 < 75	CM
CM_MB/HC20	80	20	300 < 75	CM
FDM_MB	100	0	-	FDM
FDM_MB/HC5	95	5	300 < 75	FDM
FDM_MB/HC10	90	10	300 < 75	FDM
FDM_MB/HC15	85	15	300 < 75	FDM

**Table 3:** Elastic modulus (E), tensile strength (TS) and elongation at break (EB) of sample obtained by compression molding and FDM.

Sample	E (MPa)	TS (MPa)	EB (%)
CM_MB	74.3 ± 0.84	18.6 ± 0.5	821 ± 1.8
CM_MB/HC5	121 ± 11.3	23.7 ± 2.33	43.6 ± 3.39
CM_MB/HC10	131 ± 8	24.5 ± 2.16	39.8 ± 2.75
CM_MB/HC15	145 ± 12	27.2 ± 0.88	24 ± 0.88
CM_MB/HC20	236 ± 8.49	33.4 ± 0.29	20.3 ± 0.37
FDM_MB	83.9 ± 1.34	27.2 ± 0.16	58.2 ± 0.75
FDM_MB/HC5	166 ± 8.8	41.5 ± 1.39	42.3 ± 4.19
FDM_MB/HC10	188 ± 1.54	45 ± 1.36	34.6 ± 0.82
FDM_MB/HC15	-	-	-
FDM_MB/HC20	-	-	-

## Data Availability

The data presented in this study are available on request from the corresponding authors.
